# EXAMINING THE ROLE OF AGE STEREOTYPES FOR DEVELOPMENT IN CONTEXT

**DOI:** 10.1093/geroni/igad104.0891

**Published:** 2023-12-21

**Authors:** M Clara P de Paula Couto, Maria Wirth

## Abstract

This session will focus on age stereotypes, that is, beliefs individuals hold about older adults and the aging process. Two types of age stereotypes will be addressed: those related to how older adults are assumed to be (i.e., descriptive stereotypes) and those related to how older adults should be (i.e., prescriptive stereotypes). Age stereotypes have been shown to be incorporated into older adults’ self-concepts (via stereotype internalization) and to influence their development in old age by shaping their expectations, motivations, and behaviors (stereotype embodiment). Considering the relevance of age stereotypes for developmental outcomes, the talks in this symposium will address how they shape individual aging and to what extent age stereotypes and their effects on development are context dependent. The first two presentations focus on cross-cultural differences in age stereotypes. de Paula Couto et al. examine prescriptive age stereotypes across different countries and investigate underlying factors that may influence their differential endorsement. Fung and Fung investigate cultural and age-related differences in the internalization of age stereotypes. The developmental consequences of negative age stereotypes will be addressed by Kornadt et al., whereas their malleability and specificity will be examined by Wirth et al. The last talk by Degen et al. will focus on the fear of loneliness in older adults, and how it affects their motivation to engage in behaviors that can prevent being lonely in old age.

